# Therapeutic strategies of different HPV status in Head and Neck Squamous Cell Carcinoma

**DOI:** 10.7150/ijbs.58077

**Published:** 2021-03-10

**Authors:** Yingming Sun, Zhe Wang, Sufang Qiu, Ruoyu Wang

**Affiliations:** 1 Department of Radiation and Medical Oncology, Affiliated Sanming First Hospital of Fujian Medical University, Sanming 365001, P. R. China.; 2 Department of Medical Oncology, Affiliated Zhongshan Hospital of Dalian University, Dalian 116001, P. R. China.; 3 The Key Laboratory of Biomarker High Throughput Screening and Target Translation of Breast and Gastrointestinal Tumor, Dalian University, Dalian 116001, P. R. China.; 4 Department of Radiation Oncology, Fujian Medical University Cancer Hospital & Fujian Cancer Hospital; Fujian Provincial Key Laboratory of Translational Cancer Medicine, Fuzhou 350014, P.R. China.

**Keywords:** HPV, HNSCC, PI3K, P53, immunotherapy

## Abstract

Head and neck squamous cell carcinoma (HNSCC) is the 9^th^ most common malignant tumor in the world. Based on the etiology, HNSCC has two main subtypes: human papillomavirus (HPV) -related and HPV-unrelated. HPV-positive HNSCC is more sensitive to treatment with favorable survival. Due to the different biological behaviors, individual therapy is necessary and urgently required to deduce the therapeutic intensity of HPV-positive disease and look for a more effective and toxicity-acceptable regimen for HPV-negative disease. EGFR amplification and PI3K/AKT/mTOR pathway aberrant activation are quite common in HPV-positive HNSCC. Besides, HPV infection alters immune cell infiltrating in HNSCC and encompasses a diverse and heterogeneous landscape with more immune infiltration. On the other hand, the chance of HPV-negative cancers harboring mutation on the P53 gene is significantly higher than that of HPV-positive disease. This review focuses on the updated preclinical and clinical data of HPV-positive and HPV-negative HNSCC and discusses the therapeutic strategies of different HPV status in HNSCC.

## Introduction

Head and neck squamous cell carcinoma, including those of the lip and oral cavity, nasal cavity, paranasal sinuses, oropharynx, larynx, and nasopharynx, is the 9^th^ most common malignant tumor in the world, representing 6% of all cancer cases and up to 2% of all cancer-related deaths 1, 2. HNSCC is a biologically diverse and genetically heterogeneous disease. Smoking, betel nut, and alcohol consumption are the traditional high-risk factors for HNSCC 3-7. During the past three decades, people realized human papillomavirus (HPV) 1, 8, 9 and Epstein Barr Virus (EBV) 10-12 are associated with the development of HNSCC.

Based on the etiology, HNSCC has two main subtypes: human papillomavirus-related (HPV-positive) and human papillomavirus-unrelated (HPV-negative). The HPV-positive disease occurs predominantly in the oropharynx 13. In contrast, HPV-negative disease is driven by chemical mutagenesis associated with tobacco and alcohol exposure and origins in anatomic sites 14. HPV infection transforms cells in tonsillar crypts and boosts carcinogenesis in the oropharynx 15. HPV-positive HNSCC tending to arise in a younger patient population is more sensitive to treatment with more favorable survival. Overall survival rates at 3 years are estimated at 82% in locally advanced HPV-positive HNSCC compared to 57% for locally advanced HPV-negative HNSCC 1, 9, 16-19. More preclinical and clinical data recommends a different therapeutic strategy for HPV-positive and HPV-negative HNSCC as two clinically distinct diseases 20. Recently, many clinical trials design for HNSCC based on HPV status (Table 1). As for the different biological behaviors, individual therapy is necessary and urgently required for deducing the therapeutic intensity of HPV-positive disease and looks for a more effective and toxicity-acceptable regimen for HPV-negative disease 19, 21. This review summarizes the up-to-date research and describes the emerging therapeutic strategies on HPV biased HNSCCs.

## HPV status and chemoradiotherapy

### HPV has prognostic but not predictive effectiveness of chemoradiotherapy

Radiotherapy and chemotherapy are critical therapeutic ways to treat HNSCC, and they are even the gold standard for patients with local advanced stage or recurrence and metastasis disease. RTOG 0129, the most cited trial, reported that HPV-positive patients had better 3-year rates of overall survival (82.4%, vs 57.1%; *p*<0.001) 22-25. Many scholars concluded that the clinical outcomes after chemoradiotherapy were similar between HPV+ and HPV- cohort 16, 17, 20, 25. Retrospective sub-analyses in randomized trials failed to conclude a benefit from HPV status: in the DAHANCA 5 study, nimorazole with radiotherapy has more effect in the p16-positive cohort 26. However, the TROG 0202 phase III trial presented a trend favoring the tirapazamine arm in p16-negative patients 27, 28. In conclusion, the present data in HNSCC suggest that HPV (p16) has prognostic but not predictive effectiveness of chemoradiotherapy.

### HPV-associate disease may not receive aggressive chemoradiotherapy

The clinical significance of HPV status indicates the prognosis but cannot change the clinical decision-making. According to the guidelines of NCCN 2020, surgery or radiotherapy alone can be considered for HPV-positive oropharyngeal carcinoma in the T1-2 N0 stage, surgery, radiotherapy, or concurrent radiotherapy and chemotherapy can be regarded as in T1-2N1 (lymph nodes smaller than 3cm), while tumors in a later stage can consider surgery, concurrent radiotherapy, and chemotherapy or sequential radiotherapy and chemotherapy after induced chemotherapy. For radical radiotherapy, the equivalent biological dose (EQD2) is still required to 70Gy or above (National Comprehensive Cancer Network. Head and Neck Cancers (Version 1.2021). https://www.nccn.org. Accessed December 27, 2020).

However, because of the treatment sensitivity of HPV-positive oropharyngeal carcinoma, appropriately reducing the treatment intensity would be an ideal strategy 29. Therefore, in recent years, studies on intensity reduction therapy for HPV-positive oropharyngeal carcinoma emerge one after another. In 2018, ECOG1308 suggested that local advanced HPV-positive oropharyngeal cancer with complete remission after induced chemotherapy can safely reduce the radiation dose to 54Gy 30. OPTIMA is a phase II trial to explore different radiotherapy doses according to recurrence risk. Risk-stratified dose and volume de-escalated RT/CRT are associated with favorable outcomes and less acute and chronic toxicity for HPV-positive oropharyngeal cancer 31. In 2020, ASCO also updated the data of ECOG 3311. The moderate-risk group were randomized and received standard adjuvant dose (60 Gy) radiotherapy reduction therapy (50 Gy). 2 years PFS was similar in the two groups 32.

Reducing the intensity of chemotherapy is another strategy for HPV-positive disease. RTOG 1016 indicates that cetuximab has no merit to improve prognosis 33. In the same period, two other studies with a similar design, De-ESCALaTE 34 and TROG 12.01 35, also achieved negative results, suggesting that cisplatin should still be used as the standard treatment. Many ongoing trials are going on to uncover the results; however, the treatment of HPV negative HNSCC in clinical practice needs more exploration.

## EGFR/PI3K/AKT/mTOR pathway might be potentially targeted to HPV-associated HNSCC

### EGFR/PI3K/AKT/mTOR pathway aberrant activation frequently occurs in HPV-positive HNSCC

As described above, HPV status contributes less to conventional treatment. As we know, cancer derives through the accumulation of genetic and epigenetic alteration in genes involved in a variety of signaling pathways and precipitates the cancer-associated phenotypes 29, 36.

### EGFR blockade is not a good choice for HPV-positive HNSCC

About 70% of HNSCC has an epidermal growth factor receptor (EGFR) overexpression. Hwang et al. reported that E5 forms a complex with the EGFR and triggers the EGFR pathway activation 37. Also, E5 binds the 16 kDa subunit C of the V-H+-ATPase inhibits the degradation of EGFR. c-Cbl as a ubiquitin ligase associates with the activated EGFR and targets it for degradation was hijacked by E5 38-40. To sum up, E5 is essential to trigger the EGFR pathway.

Decades ago, scholars initiated to block EGFR 41-45 on HNSCC. Monoclonal antibodies and inhibitors of EGFR had been developed, including cetuximab, afatinib, and panitumumab. A retrospective analysis from the SPECTRUM trial 46 indicated that panitumumab's therapeutic benefit was restricted to the p16 negative patients. A small sample from the PRISM trial suggested that patients with p16 negative trends benefit from panitumumab 47. Afatinib, the 2^nd^ generation of EGFR-TKI, is more pronounced in p16 negative tumors 48, 49. The EXTREME trial was the soundest in HNSCC recurrent/metastatic disease and showed that patients with p16 positive would have more benefit from cetuximab 50, 51. However, more studies regarding anti-EGFR, including the PARTNER study 52 and some other small sample trials, showed that HPV status failed to differentiate the EGFR blockade 48, 53, 54. In 2015, Seiwert et al. analyzed the TCGA HNSCC samples and showed fewer EGFR aberrations in HPV-negative tumors in genomic analyses 55. Thus, many scholars agree that EGFR expression or amplification may not be a predictive factor for EGFR blockade therapy.

### HPV trigger PI3K/AKT/mTOR pathway in various ways

The heterogeneity of EGFR blockade therapy, in part, because of the downstream driver gene mutations, remains an incomplete understanding. PI3K mutation is highlighted in HPV-associated HNSCC. In the TCGA database, 27.8% PI3K mutated in HPV positive cases 55. Vivian et al. analyzed the GWAS data from 151 HNSCC cases and determined the PI3K was the most frequently mutated (30.5%), and in HPV-associated tumors, only PIK3R1 (453_454insN), PIK3CA (E542K), and PIK3CA (H1047L) were identified 56. PI3K pathogenic mutation is relevant to advanced disease, suggesting that PI3K fuels the progress of HPV-associated HNSCC 56-59. However, the PIK3CA mutations are not the only genetic alterations that maintain activation of PI3K and downstream targets, including AKT and mTOR, in HNSCC 60-62. Indeed, 80-90% of HNSCC gain an abnormal activation of the PI3K/AKT/mTOR pathway, indicating that multiple steps of genetic and epigenetic alteration may involve the carcinogenesis which PI3K drives. Downstream signaling genes of PI3K, AKT2, mTOR, TSC1, and TSC2 were less mutated (<2%) 13, 55, 56, 59, 61.

**Figure 1:**
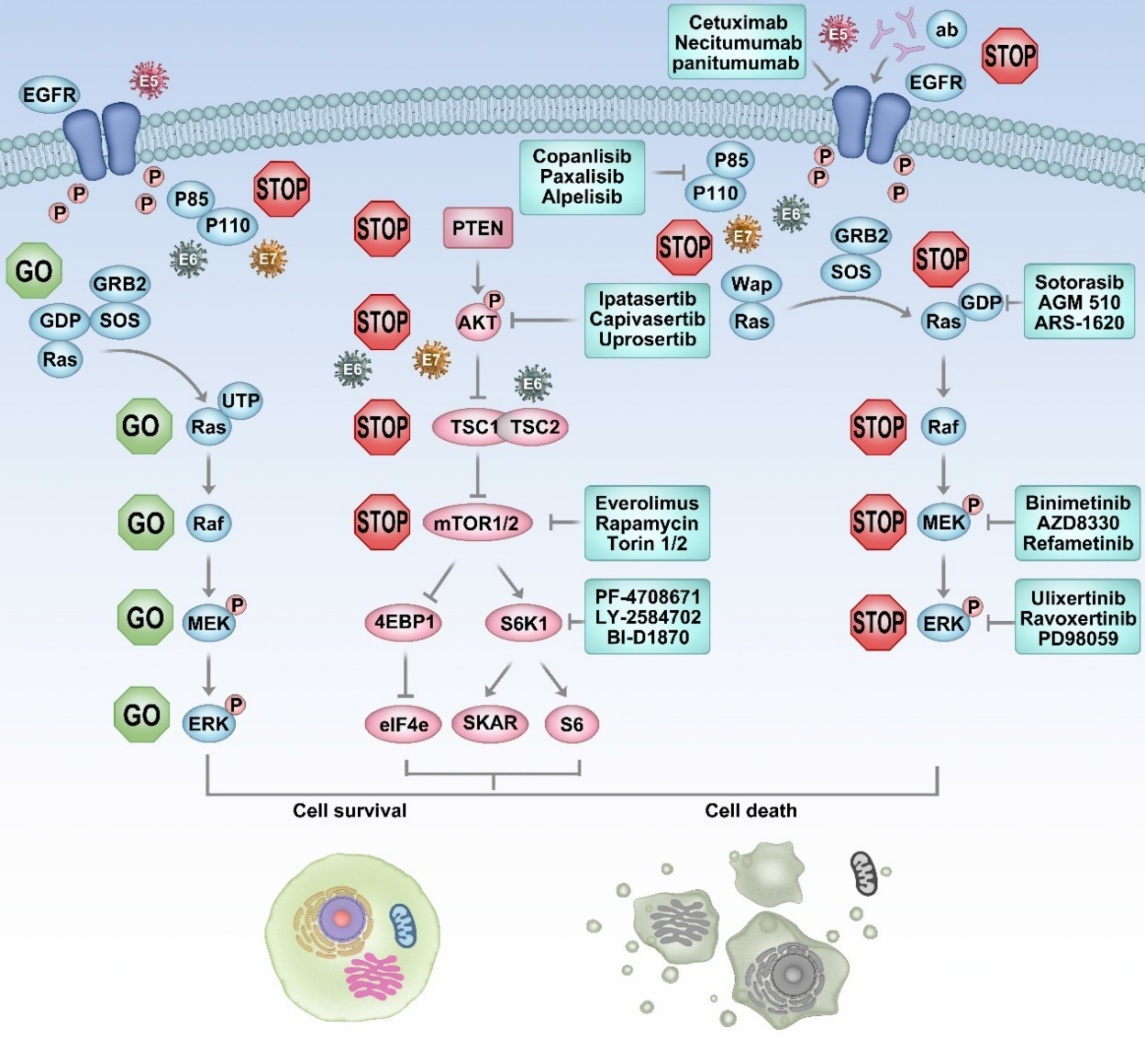
** EGFR/PI3K/AKT/mTOR pathway might be potentially targeted to HPV-associated HNSCC.** HPV E5, E6, E7 can activate PI3K/AKT/mTOR in various ways. The heterogeneity of the EGFR blockade is attributed to, somehow, the downstream driver gene mutation. About 80-90% of HPV-associated HNSCC has PI3K/AKT/mTOR pathway activation. Monotherapy of PI3K/AKT/mTOR is less effective. PI3K and MAPK pathways conduct in parallel. Combine PI3K with MAPK inhibitors in patients where genetic alterations coexist in both pathways that may induce a synthetic lethal.

Aside from the PI3K pathway's stromal mutations, many studies reveal that E6 can target phosphorylase and activate the pathway. HPV E6 oncoprotein contains a PDZ-binding domain and inactivates PTEN 62-65, leading to fuel pAkt 66, 67. Also, E6 and BPV1 interact with acidic LxxLL motifs and activate mTOR 68. E6‐E6AP complex binds and degrades the TSC2 69. E6 can degrade the NHERF-1 and activate the PI3K/Akt pathway 70. The E7 protein is known as Rb disruption and results in dysfunction of cell cycle checkpoints that promotes carcinogenesis 71-73. Loss-of-function of Rb induces AKT activation, and E7 can directly phosphorylate Akt at Thr 308 and Ser 473 74, 75. Also, E7 can bind to the ubiquitous and conserved serine/threonine phosphatase, which is crucial to protect AKT dephosphorylation and sustain AKT activation 76 (Figure 1).

### PI3K inhibitors are promising but still have a long way to go

Many pan-class I PI3K inhibitors have been developed and tested. The safety profile of inhibitors is acceptable 77. Single-agent PI3K inhibitor does not present a potent effect like EGFR-TKI and works on the selected tumor 77-80. FDA only approves alpelisib for breast cancer in PI3K mutated breast cancer and paxalisib for DIPG. Alpelisib monotherapy is unavailable in all breast cancer, and it needs to combine with fulvestrant for its therapeutic effect 81, 82. Paxalisib prolongs 5 months overall survival (OS) than TMZ, independent of PI3K mutation status 83, 84. In HNSCC, only BKM120 monotherapy was applied in the clinical trial (NCT01737450).

Herein, finding a reasonable mode of combined therapy is the best choice for targeting PI3K. As the same as PI3K inhibitor, mTOR inhibitors often present a short-term efficacy 85. Preclinical and clinical studies indicate mTOR blockade results in PI3K and Akt's reactivation via various negative feedback blockade 86-88. Dactolisib, a PI3K/mTOR dual inhibitor, exhibits a therapeutic effect superior to everolimus in the mouse model. However, excessive toxicity in patients restrains Dactolisib for pharmaceutical use 89, 90. MAPK pathway and interpathway inhibition should be explored.

### Synthetic lethal with PI3K inhibitors may be highlighted for HPV-positive HNSCC

It is well known that PI3K and MAPK pathways conduct in parallel. Combine PI3K with MAPK inhibitors in patients whose genetic alterations in both pathways coexist 58, 91-93. Scholars note that PIK3CA mutations often coexist with KRAS and BRAF mutations 94. A parallel oncogenic pathway activation abrogates the effects of PI3K/Akt/mTOR inhibitors 77, 79. Vemurafenib and dabrafenib are two successful drugs targeting BRAF; however, they present a very modest effect and only last 6 months PFS 95-98. Vitro drug screening shows that PI3K pathway aberrant activation may contribute to BRAF inhibitor resistance 99. As the same, the MEK inhibitor presents the same phenotype. Drugs or RNA interfere, targeting PI3K rescues the resistance to BRAF and MEK inhibitors. An animal model double-agent of PI3K and MEK pathway inhibitor shows a strong therapeutic effect 94, 100, 101. Unfortunately, a Phase Ib study of the pan-PI3K inhibitor Buparlisib combined with the MEK1/2 inhibitor Trametinib meets the problems of frequent dose interruptions and reductions for toxicity ease before Phase II trial 102. In 2018, novel MEK inhibitor pimasertib and PI3K/mTOR inhibitor voxtalisib also failed due to long-term tolerability and limited anti-tumor activity 103. EMR 20006-012 is another trial to evaluate pimasertib (MEK inhibitor) with SAR245409 (PI3K inhibitor) and terminates early due to low ORR and high rate of discontinuation 104. MEK inhibitor trametinib and the AKT inhibitor GSK2141795 combination strategy lunched in cervical cancer; AML also terminated due to the high rate of discontinuation or lack of clinical efficacy 105, 106. It seems that Ras mutation status does not affect the efficacy of the dual blockade of PI3K and MAPK.

It has been reported that activation might be a prognostic marker for radiotherapy in HNSCC and indicates PI3K pathway inhibition would potentially exert a synergistic effect with radiotherapy 62. In 2011, NCCTG N057K showed a well-tolerance of the combination of everolimus and radiotherapy; however, the group updated their result in 2015, and everolimus did not change the clinical outcome 107. A phase Ib clinical trial indicates a novel PI3K inhibitor Alpelisib at 250 mg/d combined with cetuximab and IMRT is tolerable and presents a clinical efficacy in local advanced HNSCC 108. Furthermore, some PI3K inhibitors, such as voxtalisib 103, 109, nelfinavir 110, 111, and buparlisib 112, also present a favorable safety profile with radiotherapy, but the clinical efficacy needs further investigation (Table 2).

## Targeting P53 is an effective strategy to fight against HPV-negative HNSCC

### Directly targeting P53 is good but hard to fight

Many trials have proved a worse clinical outcome in HPV-negative HNSCC. In RTOG 0129 trial, 433 patients with oropharyngeal cancer received cisplatin with radiotherapy. Compared with HPV-positive patients, the OS of HPV-positive tumor patients was remarkably worse (the 8-year survival rate was 71% vs 30%, HR 0.34, 95%CI 0.22-0.52). All the present data indicate that patients with HPV-negative tumors should be considered high-risk, and intensive, multimodal therapy is needed to avoid compromising their survival 22, 23, 25.

Genomic alterations are very common in HNSCC. HPV-negative cancers harbor significantly more mutations in the P53 gene than HPV-positive disease; meanwhile, loss-of-function variants in P53 are almost low to none in HPV-associated HNSCC 13, 55. In HPV-positive HNSCC, E6 promotes the MDM2-independent degradation of P53. Inactivation variants of P53 driven by tobacco may not be the same as the degradation of P53 by HPV E6 113, 114. Therefore, HPV-positive HNSCC may contain full-of-function p53.

As a transcription factor, P53 plays a crucial role in downstream target gene transcription. Under the stress of DNA damage, proto-oncogene activation, hypoxia, and microtubule damage, P53 is activated in the process of signal transduction. As a result, cellular behaviors, such as cell cycle arrest, apoptosis, aging, and angiogenesis inhibition, are performed to keep the benignancy of the cells'. P53 repairs the abnormal chromosome distribution in cells after DNA damage and mitosis 113, 115, 116. However, the anti-tumor route of targeting the P53 strategy is not easy in the past 50 years.

Decades ago, to overcome the loss function of mutated P53, people tried to reintroduce wild type P53 into the tumor via adenoviruses as vectors, and the virus can then selectively target and kill cancer cells. Adenovirus is safe and should be an ideal carrier for gene therapy as it can infect both mitoses and quiescent cells, and the genome keeps episomal and does not integrate into the host cells 117, 118. Studies have shown that the introduction of the Ad-p53 gene into P53 mutated HNSCC cells can increase tumor cells' radiosensitivity 119. Moreover, the Ad-p53 virus is active *in vivo* and compromise tumor growth in the HNSCC animal model. INGN 201 is the first candidate of Ad-p53 for the Phase I clinical trial. The study recruited 33 patients with recurrent HNSCC and received INGN 201 intratumor injection. In the resectable cohort, 27% of HNSCC patients remained disease-free, with a median follow-up period of 18 months after surgery. Of the 17 unresectable diseases, in 2 cases had PR, 6 cases kept an SD, and 9 cases evaluated as PD. Multiple direct intratumor injection courses of INGN 201 were well tolerated, and no dose-limiting toxicity or serious adverse events were observed 120-122. ONYX-015 is a chimeric virus that consists of 2 species of C adenovirus genomes, serotypes 5 (Ad5) and serotype 2 (Ad2). ONYX-015 was designed to efficiently replicate in and lyse p53-deficient cells while not affect cells with wild-type p53 123. In a Phase II trial in recurrent or refractory HNSCC patients to evaluate the safety and effect of ONYX-015, in the hyper-dose group, 1 patient had CR, 4 patients had SD, and 2 patients had PD, there was no severe AEs observed 124, 125. Besides, the effect of ONYX-015 was dependent on the mutant p53 status.

Loss-of-function P53 mutation is mainly located in the DNA binding domain, which effectively prevents the mutant p53 from binding to the target gene's response elements. p53-reactivating small molecules, including CP-31398 and PRIMA-1, are developed and tested in HNSCC 126. Roh et al. report that both CP-31398 and PRIMA-1 can attenuate HNSCC cells' proliferation and exert a synergetic effect with chemotherapy agents 127. RITA, another p53-Reactivating small molecule, directly disrupts the interaction between p53 and MDM2 and presents a potent anti-tumor impact on HNSCC, and enhances the sensitivity of cisplatin in HNSCC cells 128. However, the cell often obtains the resistance profile after directly targeting P53 treatment very soon. Adenoviral therapeutic strategies, such as Ad-p53 and ONYX-015, and small molecular compounds, have progressed to clinical trials in the HNSCC but have shown a very mild activity 113, 122, 126, 127. More and more trials list in Table 2 are ongoing to figure out the best candidate or partner of P53 RAs.

### DNA damage responders and P53 mutation may exert synthetic lethality in HPV-negative HNSCC

Due to the PARP inhibitor's great success, scholars move their focus on hunting the synthetic lethal genes of P53. There is a complicated synthetic lethal relationship between mutated p53 and its corresponding target genes in all kinds of tumors. Based on the various research results of p53 synthetic lethality, the corresponding synthetic lethal relationship can be explained from two aspects: periodic regulatory genes and aperiodic regulatory genes 129, 130.

**Figure 2:**
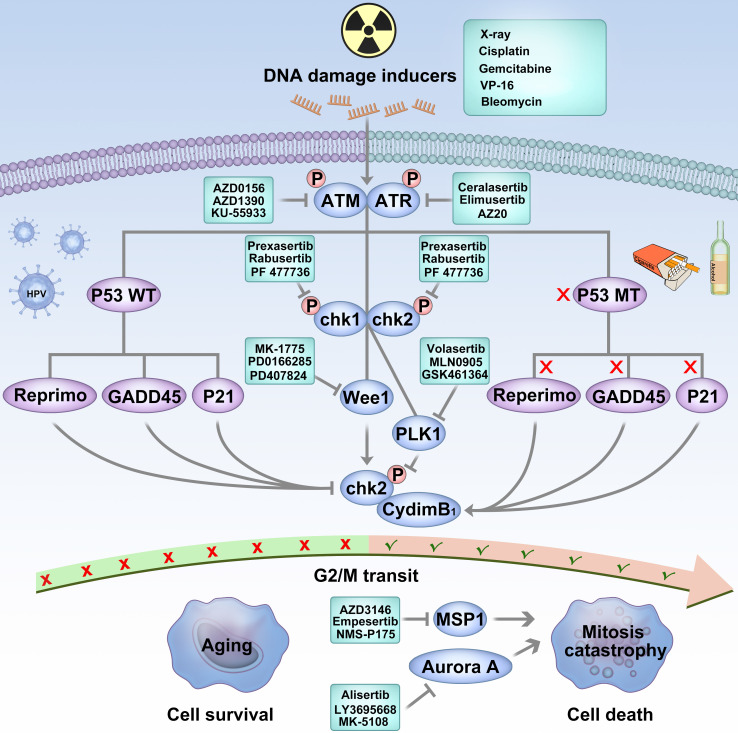
** Targeting P53 is an effective strategy to fight against HPV-negative HNSCC.** HPV-negative cancers harbor a mutation in the P53 gene as a result of tobacco and alcohol consumption. DNA damage occurs in p53 wild-type cells; the cell cycle checkpoints of G1 and G2/M are activated, which prevents the accumulation of DNA damage and may induce senescence. DNA damage repair carries out cell cycle arrest and initiates by acting on G1 and G2/M cell cycle checkpoints. In p53 mutated tumor cells, due to the lack of G1 checkpoint regulated by p53, it is more dependent on the G2-M cell cycle checkpoint when DNA is damaged. When p53 mutated cells are injured by chemotherapy or radiotherapy, blocking the G2/M cell cycle checkpoint push the unrepaired chromosome into M phase and result in a mitosis catastrophe.

It is well known that p53 activation can lead to cell cycle arrest and initiate DNA repair in response to DNA damage. Key protein inhibitors that regulate DNA damage response and cell cycle progression have the potential to be synthetic lethal partners 115. As shown in Figure 2, targeting ataxia-telangiectasia mutant (ATM), ATM-Rad3-associated (ATR), DNA-dependent protein kinase (DNA-PK), checkpoint kinase-1/2 (Chk1/2), and Wee1 kinase shows up-and-coming prospects to overcome mutant P53 HNSCC 130.

ATM and ATR are members of the phosphatidylinositol 3-kinase-related kinase (PIKK) family of serine/threonine protein kinases. ATM and ATR are two critical factors to initiate the DNA damage response as a DNA damage sensor. ATM and ATR act as primary regulators of double-strand breaks and DNA replication stress, respectively. These two kinases perform functionally overlapping but non-redundant activities 131, 132. We have a strong rationale to explore ATR inhibitors' anti-tumor efficacy in p53-mutated tumors within certain clinical settings. The dual loss of ATR and p53 function in adult mice is demonstrated to lead to defective hair follicle and tissue regeneration 133. In cancer cells treated with DNA-damaging agents, synergistic effects between ATR and TP53 have also been observed. Dual inhibitory of TP53 and ATR comes down in global loss of DNA damage checkpoints and exert a synthetic lethality 134. As a DBS response sensor, the preclinical data has presented a potent synergistic effect when an ATM inhibitor combines with radiotherapy in P53 mutated cells 135. Currently, the small-molecule inhibitors of ATR, ATM are developed and progresses to I/II phase clinical trial.

As major regulators of the ATM/ATR pathway, Chk1 and Chk2 kinases are functionally overlapping activated in response to DNA replication stress, cell cycle progression, chromatin remodeling, and apoptosis 136, 137. Basic research indicates that Chk1/2 faithfully regulates DNA repair and replication when P53 deficiency occurs 134. Gadhikar et al. used Chk1/2 inhibitor AZD7762 in mutant p53 OSCC cells. Due to the out-of-control G2/M checkpoint, AZD7762 and cisplatin pushed the cell into a mitotic catastrophe 138. Several early phase clinical trials on Chk1/2 inhibitors have been conducted in HNSCC.

When DNA damage occurred in P53 wild type cells, the cell cycle checkpoints of G1 and G2/M are activated and prevents the accumulation of DNA damage. In P53 mutated cells, due to the dysfunction of the G/1 checkpoint, the G2/M cell cycle checkpoint is crucial when DNA is damaged 115, 116. In mutated p53 cells, DNA damage repair is predominantly regulated by Wee1. It can mediate the activation of the G2/M cell cycle checkpoint, inhibit the phosphorylation of cyclin-dependent kinase 1 (Cyclin-dependent kinase 1, CDK1), and block the progression of the tumor cell cycle. Thus, the Wee1 kinase inhibitory may sensitize p53 mutant cancer cells to DNA-damaging therapy 139, 140. Many articles have proved that AZD1775, a selective and potent wee1 inhibitor, abrogated the G2 checkpoint and selectively sensitized p53 mutant cancer cells to DNA-damaging inducers, such as gemcitabine 141, cisplatin 142, and X-ray 143. Considering these findings, AZD1775 has been tested in a phase I/II clinical trial in patients with advanced solid tumors and showed well-tolerance and promising therapeutic effects 144, 145. Osman et al. used AZD1775 in HPV-negative HNSCC cells and reversed the cisplatin resistance 138. Intriguingly, Tanaka et al. reported that AZD1775 monotherapy potentiates the cisplatin response of HPV-positive HNSCC cells. Unlike HPV-negative OSCC cells, AZD1775 induces apoptosis triggered by selective cleavage of the antiapoptotic proteins MCl-1 and XIAP 146. Busch et al. revealed that the wee1 and chk1 blockade enhanced the radiation sensitivity in HPV-positive cells, and FOXMI could be a catalyst between AZD1775 and X-ray HPV-positive cells 147, 148. However, most present evidence based on basic research requires further clinical trials to investigate the safety and effect of AZD1775 further. Table 3 listed all the clinical trials targeting P53 and DNA damage responder inhibitors in HNSCC.

## HPV-associated disease trend to obtain more benefit from immunotherapy

### Landscape of microenvironment of different HPV-status HNSCC

Tumor-infiltrating lymphocytes are associated with improved prognosis. HNSCC arises from squamous epithelium associated with the tongue's tonsils and base and is deemed to have more immune cells infiltrated within the tumor microenvironment. HPV infection alters immune cell, which infiltrates in HNSCC to encompass diverse and heterogeneous landscapes 36.

HPV viral factors, E5, E6, and E7, play a crucial role in generating an immunosuppressive microenvironment that promotes tumor progression. Oncoprotein E5 blocks HLA-C and HLA-E from tumor stroma to interact with MHC I on the cancer cell, impairing T cell and NK cells' activity. 149. Moreover, E5 attenuates MHC class II's expression and stability by blocking peptide loading and transportation. By interfering with MHC, E5 may severely impair antigen processing and T cell activation 150. In the past 20 years, scholars get extensive knowledge about E6 and E7. The mechanism of E6 and E7 to regulate the tumor microenvironment is not linear. Briefly, in 2012, Vandermark et al. reported that E6 and E7 proteins alter the NF-kB pathway in tumor cells, impair the innate immunosystem, and evade supervision 151. E6 and E7 can interact with keratinocytes and inhibit macrophage infiltration by decreasing the secretion of MIP-3α 152. IL-10 is a double-edged sword, and it can exert both pro-tumor and anti-tumor effects, including hiding the MHC, repression of DCs, and activation of NK and T cells. Poved et al. reported, HPV E6 and E7 can bind to the promoter of IL10 and enhance the expression of IL10. What is more, IL10 can promote the expression of E6 and E7 that create positive feedback for an immunosuppressive environment 153, 154. Toll-like receptor 9 (TLR9) expresses on the surface of dendritic cells, macrophages, natural killer cells, and initiates the signals that cytokines' production is needed for innate and acquired immunity. HPV E7 induces a transcriptional repressor complex on the TLR9 promotor and abolishes its expression and function. HPV E6 also blocks TLR9 by NFκB pathway 155-157. Transforming growth factor beta (TGF-β) is a multifunctional cytokine, which can regulate the inflammation process of tumor, polarize the macrophagy and stimulate the myeloid-derived suppressor cell (MDSC) 158. E6 and E7 enable to activate the TGF-β promoter throughout the Sp1 recognition sequence 159. E5 and E7 play different role in PD1/PDL1 axis. Briefly, E5 mediates resistance to PD-L1 Blockade by hiding the MHC 160. Chaoqi Liu exogenously expressed E7 on PC3 cells and led to a PDL1 increase 161. Furthermore, HPV E7 also can regulate IDO 162, CXCL14 163, and c-GAS-STING 164 to generate an immunosuppressive microenviroment.

**Figure 3:**
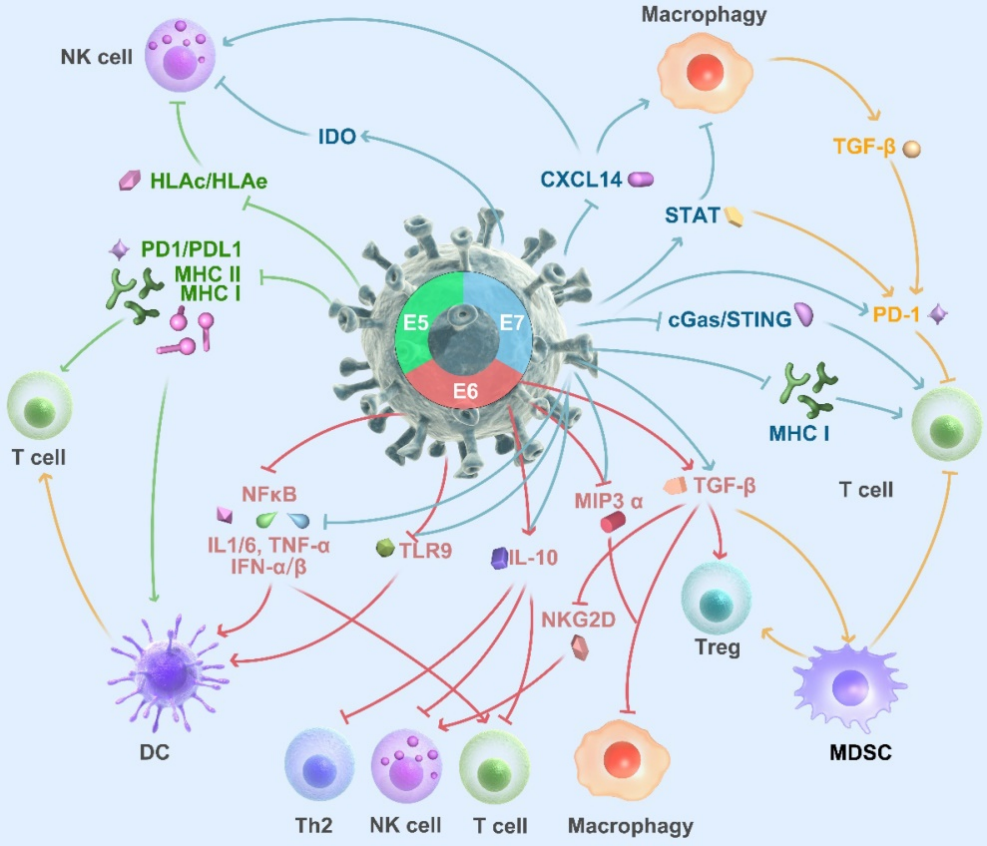
** HPV infection alters immune cell infiltrates in HNSCC.** HPV E5, ER6, E7 module HNSCC microenvironment. Briefly, E5 interacts with HLA and MHCII on tumor cells to inhibit the DC and T cell activation; E6, E7 impair the NFκB pathway and regulates the immune cell infiltrate; IL-10 creates a loop with E6 and E7; E7 also regulates CXCL14 and c-GAS/STING pathway to modulate the immune cell infiltration.

Mandal et al. reported in an analysis of TCGA found that HPV-positive HNSCCs had more significant immune infiltration and had higher T cell levels with granzyme and perforin 165. Tregs and NK cells were also frequently infiltrated in HPV positive tissues and associated with a favorable prognosis 166, 167. Wood et al. identified increased expression of genes encoding PD1, CTLA-4, and TIM-3 in HPV-positive HNSCC 168, which suggests patients with HPV-positive status may obtain more benefit from immune checkpoints blockade (Figure 3).

### Promising but not sure, HPV-positive HNSCC could obtain more benefit from immunotherapy

Promising clinical trial results led to pembrolizumab's FDA approval for treating refractory or metastatic HNSCC in 2016. Keynote 012 study was the first clinical trial to evaluate the relationship between HPV status and immune checkpoints blockade response. 60 patients with PD-L1+ (> 1%) were treated with pembrolizumab. ORR was 24% (95% CI, 13-40%) in those patients with HPV positive, and 16% in HPV negative patients (95% CI, 10-23%, P > 0.05) 169.

In the phase III Checkmate 141 study, nivolumab obtained a better OS than standard treatment (median OS: 7.5 months vs 5.1 months, HR: 0.70; 97.73% CI, 0.51-0.96; P=0.01), with an approximately 19% increase for 1-year survival. In the Checkmate 141 subgroup analysis, patients with p16 positive tumors tend to obtain more benefit from nivolumab than the standard treatment group (median OS: 9.1 months vs. 4.4 months HR: 0.56; 95% CI: 0.32 to 0.99). On the other hand, in patients with p16 negative disease, the median OS was 7.5 months in the nivolumab monotherapy group and 5.8 months in the standard treatment group (HR: 0.73; 95% CI: 0.42 to 1.25) 170.

However, the advantage of HPV status in nivolumab treatment failed to exhibit in pembrolizumab. In phase II KEYNOTE-055 trial, ORR was similar regardless of HPV status, with rates of 16% in HPV-associated disease and 15% in HPV-negative disease. However, medium PFS and OS did not differ based on HPV status 171. The KEYNOTE-040 trial design is the same model as checkmate 141. The median OS in the pembrolizumab group was 8.4 months, compared with 6.9 months in the standard treatment group. Although the trial failed to reach the final endpoint, the data is still promising. Patients with p16-negative disease in the pembrolizumab group had more prolonged overall survival than the standard treatment group (HR: 0.77; 95%CI: 0.61-0.97), whereas this survival benefit did not present among patients with the p16-positive disease (HR: 0.97; 95%CI: 0.63-1.49) 172-174. KEYNOTE-048 trial followed up to KEYNOTE-040; the data showed that the treatment with pembrolizumab significantly improved OS compared with the EXTREME regimen in patients with PD-L1 expression (CPS ≥ 1 and CPS ≥ 20 arms). From the date, PD-L1 expression is the only reliable biomarker for HNSCC to receive pembrolizumab. While approximately 21% of patients in this study were p16-positive, they were not analyzed separately as the HPV status may not tell any difference 175.

Atezolizumab is an anti-PD-L1 mAb. In a phase I trial, 32 patients with R/M HNSCC were enrolled. The data were in line with anti-PD1 mAb. Atezolizumab monotherapy had a 22% ORR rate, mPFS of 2.6 months, and mOS of 6 months. However, this early trial's primary date displayed the effect of Atezolizumab, regardless of HPV status or PD-L1 expression level 176. Durvalumab is another anti-PDL1 and rises a Pacific storm in NSCLC. The Phase III EAGLE trial data shows the HPV-positive did not affect durvalumab 177, and patients with negative were associated with worse OS. Avelumab is a fully human anti-PD-L1. The IgG1 construction can competitively block with PD-1; however, the trial on HNSCC prematurely terminated as recommended because the boundary for futility has been crossed (NCT02952586).

Although many retrospective or bio-informative studies indicated a higher degree of T cells in curium titration in HPV-associated HNSCC, the only nivolumab showed HPV+HNSCC could obtain more benefit from ICI therapy. The present clinical data indicate that HPV status does not support strategic treatment with checkpoint inhibitors.

## Epidrugs have a long way to go in NHSCC

The imbalance, mutation, and aberrant expression of epigenetic regulatory factors boost carcinogenesis and maintain the growth and metastasis of HNSCC. Also, the levels of these epigenetic regulators varied with the type of HNSCC. From 2004, the US FDA approved only 6 epidrugs in the clinic: 2 DNMT inhibitors of azacitidine and decitabine; 4 HDAC inhibitors of Vorinostat, Romidepsin, Farydak, and Panobinostat. Many other agents are in the pipeline.

Although scientists have done much work on the epigenetic regulation of HNSCC, rare epidrugs are in clinical trials. Many studies report that HPV-HNSCC has relatively low DNA methylation levels and promotes genomic instability. Meanwhile, HPV+ HNSCC harbors distinctly hypermethylated genomes. The diversity of molecular and epigenetic between HPV+ and HPV- tumors provides a therapeutic strategy that forces the demethylation of genomes of HPV associated NHSCC.

DNMT inhibitors/DNA methylation agents present the cytotoxicity on HPV status bias. Asel Biktasova et al. reported that HPV+ HNSCC cells are sensitive to azacitidine partially due to stabilization of p53 and attenuation of the expression of HPV genes. Moreover, azacytidine is sufficient for suppressing cell invasion and sensitizing the cell to interferons. 178. However, in 2019, Ricard Mesia posted that azacitidine had an insufficient activity to further study in advanced NPC 179.

CUDC-101 is a small molecule that simultaneously blocks the EGFR, human growth factor receptor 2 (HER2), and histone deacetylase (HDAC) with promising activity in HNSCC. Thomas Galloway et al. reveals that CUDC-101 is tolerable in a phase I trial; however, a high discontinuation rate suggests CUDC-10 needs to alternate the schedules or routes of administration 180, 181. NCT02178072 is an ongoing clinical trial to evaluate CUDC-10 with Azacitidine. Asel Biktasova et al. reports that single-agent epidrug rarely presents therapeutic effects in NHSCC; most drugs cease the clinical trial before phase II 182. Oncologists move their efforts to combine epidrugs with radiotherapy or chemotherapy. Panobinostat, an HDAC inhibitor, has been identified as a tolerable drug and exerts the effectiveness of erlotinib in HNSCC 183. Valproic acid, a drug for epilepsy, has a potent effect on blocking histone deacetylase activity. A phase II clinical study showed that Valproic acid plus cisplatin and cetuximab exhibits a less toxic and more effective than standard first-line regimen in advanced HNSCC 184. Other HADC inhibitors, including Vorinostat, Belinostat, Panobinostat, present the therapeutic effect in HNSCC without HPV bias. HNSCC Oncologist is looking for an optimal combination with epidrugs, and we believe more results about epidrugs in HNSCC will be posted in the next few years; however, our point of view does not support epidrug could exert the effects on HPV bias.

To sum up, we review the conditional therapy, including chemotherapy and radiotherapy, and indicate that HPV-positive patients may obtain more benefit from traditional treatment. More clinical trials need to be investigated for the optimal dosage for HPV-positive patients. PI3K is a promising target in HPV-positive HNSCC; however, the toxicity of combination therapy with PI3K inhibitor should not be ignored. Preclinical data present a suspected result of directly targeting P53, and synthetic lethality with DNA damage responder inhibitors remains too much to be explored. Immunotherapy is effective for HNSCC regardless of HPV status. At the present stage, as the same before 5 years, the HPV status can predict the prognosis more but cannot change the clinical decision-making. More prospective clinical trials are ongoing, which may answer critical questions over the next three to five years.

## Figures and Tables

**Table 1:** Clinical trials based on HPV status in NHSCC

Clinical trial number	Project name
**HPV+**	
NCT03946358	Combination of UCPVax Vaccine and Atezolizumab for the treatment of Human Papillomavirus Positive Cancers (VolATIL)
NCT04260126	Study of PDS0101 and Pembrolizumab combination I/O in subjects with HPV16 + recurrent and/or metastatic HNSCC
NCT02048020	Paclitaxel and Carboplatin before radiation therapy with Paclitaxel in treating HPV-positive patients with Stage III-IV Oropharynx, Hypopharynx, or Larynx Cancer
NCT04444869	Testing less intensive radiation with chemotherapy to treat low-risk patients with HPV-positive Oropharyngeal Cancer
NCT03829722	Radiotherapy, Carboplatin/Paclitaxel and Nivolumab for high risk HPV-related Head and Neck Cancer
NCT03618134	Stereotactic Body Radiation Therapy and Durvalumab with or without Tremelimumab before surgery in treating participants with Human Papillomavirus positive Oropharyngeal Squamous Cell Cancer
NCT04630353	A Study HB-201 in patients with newly diagnosed HPV16+ Oropharynx or Locally Advanced Cervical Cancer
NCT01721525	Induction Chemotherapy with Afatinib, Ribavirin, and weekly Carboplatin/Paclitaxel for Stage IVA/IVB HPV associated Oropharynx Squamous Cell Cancer (OPSCC)
NCT03715946	Adjuvant de-escalated radiation + Adjuvant Nivolumab for Intermediate-high risk P16+ Oropharynx Cancer
NCT00257738	0804 GCC: MAGE-A3/HPV 16 Vaccine for Squamous Cell Carcinoma of the Head and Neck
NCT03396718	De-escalation of Adjuvant Radio (Chemo) Therapy for HPV-positive Head-neck Squamous Cell Carcinomas
NCT03578406	HPV-E6-specific anti-PD1 TCR-T cells in the treatment of HPV-positive NHSCC or Cervical Cancer
NCT04534205	A clinical trial investigating the safety, tolerability, and therapeutic effects of BNT113 in combination with Pembrolizumab versus Pembrolizumab alone for patients with a form of Head and Neck Cancer positive for Human Papilloma Virus 16 and expressing the protein PD-L1
NCT04489212	Study of Mucosal Sparing Adjuvant Radiotherapy after Surgical Exploration in HPV+ Head and Neck Cancer of Unknown Primaries
NCT03260023	Phase Ib/II of TG4001 and Avelumab in HPV16 positive R/M Cancers including Oropharyngeal SCCHN
NCT03978689	A Phase 1 Study in patients with HPV+ recurrent/metastatic Head and Neck Squamous Cell Carcinoma
NCT04369937	HPV-16 Vaccination and Pembrolizumab Plus Cisplatin for “Intermediate Risk” HPV-16-associated Head and Neck Squamous Cell Carcinoma
NCT04252248	Decitabine treatment in HPV-induced anogenital and Head and Neck Cancer patients after radiotherapy or as novel late salvage
NCT03942380	Cell-free tumor DNA in Head and Neck Cancer patients
NCT02163057	Study of HPV specific immunotherapy in patients with HPV associated Head and Neck Squamous Cell Carcinoma
**HPV-**	
NCT03944915	De-escalation therapy for Human Papillomavirus negative Disease
NCT04220749	Radiotherapy vs. Trans-Oral Surgery for HPV-negative Oropharyngeal Squamous Cell Carcinoma
NCT03356223	Evaluation of ABEMACICLIB monotherapy in patients with locally advanced/metastatic Head and Neck Cancer after failure of Platinum and Cetuximab or anti-EGFR-based therapy and harboring an Homozygous Deletion of CDKN2A, and/or an amplification of CCND1 and/or of CDK6
NCT03389477	Los Tres Paso: Neoadjuvant Palbociclib Monotherapy, Concurrent Chemoradiation Therapy, Adjuvant Palbociclib Monotherapy in patients with p16INK4a negative, HPV-unrelated Head and Neck Squamous Cell Carcinoma
NCT03635164	Radiotherapy with Durvalumab prior to surgical resection for HPV negative Squamous Cell Carcinoma
NCT04169074	Modulation of the tumor microenvironment by Abemaciclib in operable HPV-Negative Head and Neck Cancer (HNC)
NCT03673735	Maintenance Immune Check-point Inhibitor following post-operative Chemo-radiation in subjects with HPV-negative HNSCC
NCT03624231	Feasibility & Efficacy of Durvalumab+Tremelimumab+RT and Durvalumab+RT in Non-resect. Locally Advanced HPVnegativ HNSCC
NCT04247282	Anti-PD-L1/TGF-beta Trap (M7824) alone and in combination with TriAd Vaccine and N-803 for Resectable Head and Neck Squamous Cell Carcinoma not associated with Human Papillomavirus Infection

**Table 2:** Clinical trials basing on PI3K/AKT/mTOR pathway in NHSCC

Clinical trial number	Project name
NCT03740100	Single-arm study with Bimiralisib in patients with HNSCC Harboring NOTCH1 loss of function mutations
NCT02822482	Copanlisib in association with Cetuximab in patients with recurrent and/or Metastatic Head and Neck Squamous Cell Carcinomas harboring a PI3KCA mutation/amplification and/or a PTEN loss
NCT03795610	Window of Opportunity Study of IPI-549 in patients with locally advanced HPV+ and HPV- Head and Neck Squamous Cell Carcinoma
NCT04193293	A Study of Duvelisib in combination with Pembrolizumab in Head and Neck Cancer
NCT03356587	A Biomarker-driven, Open Label, Single Arm, Multicentre Phase II Study of Abemaciclib in patients with recurrent or metastatic Head and Neck Squamous Cell Carcinoma who failed to Platinum-based Therapy
NCT01602315	A Phase Ib/II Study of BYL719 and Cetuximab in Recurrent or Metastatic Head and Neck Squamous Cell Carcinoma
NCT02537223	Phase I Study of BYL719 in combination with Cisplatin and Radiotherapy in patients with Squamous Cell Head and Neck Cancer
NCT02573493	Nab-Paclitaxel and Cisplatin or Nab-paclitaxel as induction therapy for locally advanced Squamous Cell Carcinoma of the Head and Neck (HNSCC)
NCT03896412	Detection of Circulating Tumor DNA in p16- Locally Advanced Head Neck Squamous Cell Carcinoma
NCT02051751	A study to evaluate the potential benefit of the addition of BYL719 to Paclitaxel in the Treatment of Breast Cancer and Head-and-neck Cancer
NCT03022409	A Study to investigate biomarker effects of pre-surgical Treatment with DNA Damage Repair (DDR) agents in patients with Head and Neck Squamous Cell Carcinoma (HNSCC).
NCT01204099	Study of PX-866 and Docetaxel in Solid Tumors
NCT01252628	Phase 1 and 2 Study of PX-866 and Cetuximab
NCT02113878	Phase Ib Study of BKM120 with Cisplatin and XRT in high risk locally advanced Squamous Cell Cancer of Head and Neck
NCT02277184	Ficlatuzumab, Cisplatin and IMRT in locally advanced Head and Neck Squamous Cell Carcinoma
NCT01816984	PI3K Inhibitor BKM120 and Cetuximab in treating patients with recurrent or metastatic Head and Neck Cancer
NCT02644122	SF1126 in recurrent or progressive SCCHN and mutations in PIK3CA Gene and/or PI-3 Kinase Pathway Genes
NCT03292250	Korean Cancer Study Group: Translational bIomarker Driven UMbrella Project for Head and Neck (TRIUMPH), Esophageal Squamous Cell Carcinoma- Part 1 (HNSCC)]
NCT02298595	Cetuximab, Cisplatin and BYL719 for HPV-associated Oropharyngeal Squamous Cell Carcinoma
NCT01349933	Akt Inhibitor MK2206 in treating patients with recurrent or metastatic Head and Neck Cancer
NCT01195922	Rapamycin Therapy in Head and Neck Squamous Cell Carcinoma
NCT01172769	Efficacy Study of Temsirolimus to Treat Head and Neck Cancer
NCT03740100	Single-arm Study with Bimiralisib in patients with HNSCC Harboring NOTCH1 loss of function mutations
NCT01111058	Everolimus versus Placebo in Head and Neck Cancer
NCT01051791	Phase II Study of RAD001 Head and Neck Cancer
NCT01016769	Temsirolimus + weekly Paclitaxel + Carboplatin for recurrent or metastatic Head and Neck Squamous Cell Cancer (HNSCC)
NCT01313390	Everolimus and Docetaxel in treating patients with recurrent, locally advanced, or metastatic Head and Neck Cancer
NCT03065062	Study of the CDK4/6 Inhibitor Palbociclib (PD-0332991) in combination with the PI3K/mTOR Inhibitor Gedatolisib (PF-05212384) for patients with Advanced Squamous Cell Lung, Pancreatic, Head & Neck and Other Solid Tumors

**Table 3:** Clinical trials targeting P53 and DNA damage responder inhibitor in NHSCC

Number	Project name
NCT02842125	Safety and Efficacy of Intra-Arterial and intra-tumoral Ad-p53 with Capecitabine (Xeloda) or Anti-PD-1 in Liver Metastases of Solid Tumors and recurrent Head and Neck Squamous Cell Cancer
NCT03544723	Safety and efficacy of p53 Gene Therapy combined with Immune Checkpoint Inhibitors in Solid Tumors.
NCT00003257	Gene Therapy in treating patients with recurrent Head and Neck Cancer
NCT02432963	Vaccine Therapy and Pembrolizumab in treating patients with Solid Tumors that have failed prior therapy
NCT00017173	S0011, Gene Therapy & Surgery Followed by Chemo & RT in Newly Diagnosed Cancer of the Mouth or Throat
NCT00404339	Vaccine Therapy in treating patients with Head and Neck Cancer
NCT00041613	Study to compare the Overall Survival of patients receiving INGN 201 (Study Drug) with patients receiving Methotrexate
NCT00041626	Effectiveness and Safety of INGN 201 in combination with Chemotherapy versus Chemotherapy Alone
NCT02567422	M6620, Cisplatin and Radiation Therapy in treating patients with locally advanced Head and Neck Squamous Cell Carcinoma
NCT04576091	Testing the Addition of an Anti-cancer Drug, BAY1895344, with Radiation Therapy to the usual Pembrolizumab Treatment for Recurrent Head and Neck Cancer
NCT03022409	A Study to Investigate Biomarker effects of pre-surgical treatment with DNA Damage Repair (DDR) agents in patients with Head and Neck Squamous Cell Carcinoma (HNSCC)
NCT01275183	Pilot Study of Raltegravir and Cisplatin in Squamous Cell Carcinoma of Head and Neck
NCT01115790	A Phase 1 study in participants with Advanced Cancer
NCT02797964	A Phase 1/2 trial of SRA737 in subjects with Advanced Cancer
NCT02508246	WEE1 Inhibitor MK-1775, Docetaxel, and Cisplatin before surgery in treating patients with Borderline Resectable Stage III-IVB Squamous Cell Carcinoma of the Head and Neck
NCT02585973	Dose-escalating AZD1775 + Concurrent Radiation + Cisplatin for Intermediate/High Risk HNSCC
NCT03028766	WEE1 inhibitor with Cisplatin and Radiotherapy: A trial in Head and Neck Cancer
NCT02196168	Cisplatin with or without WEE1 inhibitor MK-1775 in treating patients with Recurrent or Metastatic Head and Neck Cancer
